# Associations between frailty, physical performance, and renal biomarkers in older people with advanced chronic kidney disease

**DOI:** 10.1007/s41999-021-00478-4

**Published:** 2021-03-17

**Authors:** George Smith, Alison Avenell, Margaret M. Band, Geeta Hampson, Edmund J. Lamb, Roberta C. Littleford, Paul McNamee, Roy L. Soiza, Deepa Sumukadas, Miles D. Witham

**Affiliations:** 1 AGE Research Group, NIHR Newcastle Biomedical Research Centre, Newcastle University and Newcastle Upon Tyne Hospitals NHS Foundation Trust, Campus for Ageing and Vitality, Newcastle upon Tyne, NE4 5PL UK; 2 Health Services Research Unit, University of Aberdeen, Aberdeen, UK; 3 Tayside Clinical Trials Unit, University of Dundee, Dundee, UK; 4 Department of Clinical Chemistry and Metabolic Medicine, Guy’s and St Thomas’ NHS Foundation Trust, London, UK; 5 Pathology Department, East Kent Hospitals University NHS Foundation Trust, Canterbury, UK; 6 University of Queensland, Brisbane, QLD Australia; 7 Health Economics Research Unit, University of Aberdeen, Aberdeen, UK; 8 Ageing Clinical and Experimental Research, University of Aberdeen, Aberdeen, UK; 9 Department of Medicine for the Elderly, NHS Tayside, Dundee, UK

**Keywords:** Chronic kidney disease, Biomarkers, Physical performance, Frailty

## Abstract

**Purpose:**

Impaired physical performance and frailty are common in older people with advanced chronic kidney disease but it is unclear which metabolic derangements contribute to these impairments. We, therefore, examined associations between renal biochemical markers and both physical performance and frailty in older people with advanced chronic kidney disease.

**Methods:**

Secondary analysis of data from the BiCARB trial, which enrolled non-dialysing patients aged 60 and over, with chronic kidney disease stage 4/5, with serum bicarbonate < 22 mmol/L. Participants undertook the Short Physical Performance Battery, maximum grip strength and six-minute walk test at baseline, 3, 6, 12 and 24 months. Renal biochemistry (serum creatinine, cystatin C, phosphate, and bicarbonate), haemoglobin, 25-hydroxyvitamin D and NT-pro-B-type natriuretic peptide were measured at baseline. Associations between baseline renal biochemistry and physical performance, and between baseline biochemistry and the monthly rate of change in physical performance were assessed.

**Results:**

We analysed data from 300 participants (mean age 74 years; 86 [29%] women). 148 (49%) were pre-frail, 86 (29%) were frail. In multivariable cross-sectional baseline analyses, only age and BMI were significantly associated with baseline short physical performance battery; age, sex, body mass index, NT-pro-BNP and 25-hydroxyvitamin D were significantly associated with baseline six-minute walk distance. No significant associations were found between biochemical markers and change in physical performance over time, except between baseline 25-hydroxyvitamin D concentration and change in six-minute walk distance.

**Conclusions:**

Biochemical markers associated with chronic kidney disease did not consistently associate with baseline physical performance or the rate of change of physical performance measures.

**Trial Registration**: ISRCTN09486651

**Supplementary Information:**

The online version of this article (10.1007/s41999-021-00478-4) contains supplementary material, which is available to authorized users.

## Key summary points

**Aim:**

To test whether renal biochemical markers were associated with physical performance and frailty in older people with advanced chronic kidney disease.

**Findings:**

Biochemical markers associated with chronic kidney disease did not consistently associate with baseline physical performance or the rate of change of physical performance measures.

**Message:**

Targeting improvements in renal biochemistry may not be a fruitful way to improve physical function and frailty in older people with advanced chronic kidney disease.

**Supplementary Information:**

The online version of this article (10.1007/s41999-021-00478-4) contains supplementary material, which is available to authorized users.

## Introduction

Frailty is a state of impaired homeostasis and reduced physiological reserve such that a minor stressor event causes a disproportionately large decline in health status [1]. Frailty is independently associated with a range of adverse outcomes that include premature mortality, increased risk of hospital admission, longer length of hospital stay and future disability [2–4]. Impaired physical performance is a key component of the physical frailty syndrome, and measures of physical performance (low handgrip strength and low walk speed) feature in the frailty phenotype described by Fried et al. [5] along with weight loss, exhaustion and low physical activity levels. Frailty is common in patients with advanced chronic kidney disease (CKD), with a prevalence of up to 73% in those on maintenance haemodialysis [6, 7]; this compares to a prevalence of approximately 10% in the general population aged 65 and over [8]. Frailty is an independent risk factor for a range of adverse events in all stages of CKD [9].

Before interventions to prevent or improve frailty and physical performance in patients with CKD can be designed, a better understanding of the factors associated with frailty and impaired physical performance in this population is required. Several pathophysiological processes that are present in CKD could plausibly affect skeletal muscle function, thus leading to impaired physical performance and hence physical frailty. These include but are not limited to: reduction of food intake (particularly protein), persistent inflammation, oxidative stress, metabolic acidosis, anaemia, impaired vitamin D metabolism, vascular dysfunction, uraemia, and hyperphosphataemia [10–12]. These factors, together with the high prevalence of multimorbid disease that accompanies CKD [13], may all play a part in driving the high prevalence of frailty seen in patients with CKD.

The majority of studies examining the association between CKD and physical performance or frailty have been conducted with patients undergoing dialysis [9, 14]. More information is, therefore, needed on how common frailty is in patients with advanced CKD who are not on dialysis—particularly older patients who are at highest risk of frailty. In addition, many studies to date have been cross sectional in nature, limiting the ability to infer causal relationships. This analysis, therefore, first aimed to describe the prevalence of frailty in a group of older people with advanced CKD not on dialysis; second to explore associations between baseline measures of physical performance and frailty and measures of renal pathophysiology, and third to describe associations between baseline measures of renal pathophysiology and changes in physical performance during follow-up. We hypothesised that frailty would be common in older patients with advanced CKD not undergoing dialysis, that biomarkers of CKD pathophysiology would be significantly correlated with frailty and impaired physical performance at baseline and would correlate with the rate of decline in physical performance over time.

## Methods

### BiCARB trial

We analysed cross-sectional and longitudinal data collected as part of the BiCARB randomised controlled trial [15, 16], which evaluated the clinical and cost-effectiveness of oral sodium bicarbonate therapy for older patients with advanced CKD and mild metabolic acidosis. The trial was funded by the UK National Institute for Health Research (NIHR) Health Technology Assessment Board (award 10/71/01) and was co-sponsored by the University of Dundee and NHS Tayside (Tayside Academic Health Sciences Collaboration). Ethical approval was granted by the East of Scotland NHS Research Ethics Committee (approval 12/ES/0023) and the trial was approved by the UK Medicines and Healthcare Regulatory Authority (EudraCT number 2011–005,271-16; Clinical Trial Authorisation number 41692/0001/001–0001). The trial was registered at www.isrctn.com (ISRCTN09486651).

### Participants

The BiCARB trial enrolled 300 participants from nephrology and geriatric medicine clinics in 27 hospitals across the UK. Inclusion criteria were CKD stage 4 or 5 (eGFR < 30 ml/min/1.73m^2^ by MDRD4 equation [17]), not undergoing dialysis, aged ≥ 60 years, with a serum bicarbonate level at screening of < 22 mmol/L. A full list of exclusion criteria has been published previously [15]. In the BiCARB trial, administration of oral bicarbonate had no effect on measures of physical performance or other outcomes, and all participants are, therefore, included in the analyses presented here. Information on baseline cardiovascular comorbidities but not other conditions was collected.

### Outcome measures

Participants performed the Short Physical Performance Battery (SPPB) [18], a test of lower limb function that has been shown to predict a range of adverse outcomes including future disability, need for care and death [19]. The test consisted of three components—a balance test, a gait speed test, and a chair stand test. Each section was scored out of 4 points with a maximum of 12 points denoting the highest level of performance. Participants performed the six-minute walk distance (6MWD) as a submaximal test of cardiorespiratory function. Participants walked as far as possible in 6 min over a 25 m course [20] and standardised words of encouragement were delivered to the participant every 2 min. Maximal grip strength was measured using a Takei handgrip dynamometer. The maximum value of three attempts in the dominant hand was used [21]. Physical performance data were collected at baseline, 3, 6, 12 and 24 month visits and all available data were used in these analyses. A frailty score was derived using prespecified criteria based on the five domains outlined by Fried et al. [5]. A description of how the score was derived is given in the Supplementary Material. Age, sex and BMI were used as covariates.

### Baseline markers of renal biochemistry

Baseline markers of renal function (serum cystatin C, creatinine, and bicarbonate), markers associated with bone metabolism [serum phosphate and 25-hydroxyvitamin D (25OHD)], blood haemoglobin and serum albumin were measured, along with plasma NT-pro-terminal B-type natriuretic peptide (NT-pro-BNP) as a marker of cardiovascular dysfunction. Cystatin C was measured at a central laboratory using a turbidimetric immunoassay (Abbott ARCHITECT analyser, Maidenhead, UK) as previously described [16]. NT-pro-BNP was measured at a central laboratory using an enzyme-linked immunosorbent assay (Meso Scale Discovery, Rockville, MD, USA). 25OHD was analysed by a central laboratory using a Chemiflex Chemiluminescence Microparticle Immunoassay (Abbott Diagnostics, Lake Forest, IL, USA). All other blood biomarkers (creatinine, bicarbonate, phosphate, haemoglobin and albumin) were analysed by local hospital services as part of routine clinical care; all hospital laboratories participated in the UK NEQAS quality assurance scheme.

### Statistical analyses

Analyses were performed using SPSS v25 (IBM Corp, Armonk, NY) and a two-sided p value of < 0.05 was taken to be significant for all tests. We did not adjust analyses for multiple statistical testing. Data were summarised using means or medians and measures of variability were reported as standard deviation, interquartile range or 95% CI. Bivariate correlations between measures of physical performance and baseline biomarkers were derived using Pearson’s correlation coefficient or Spearman’s rho for skewed variables. Multivariable linear regression models were run using forced entry of all variables with the exception of serum phosphate, which could not be included because of high levels of missing data. Baseline measures across frailty categories were compared using independent samples t tests for normally distributed variables, or Mann–Whitney U tests for non-normally distributed variables. Categorical comparisons were conducted using Pearson’s chi-squared test (or Fisher’s exact test where any cell contained 5 or fewer cases). For each variable, non-frail was used as the referent category. The relationship between baseline cardiovascular comorbidities and physical performance measures was also described through univariate analyses. Student’s t tests were used for continuous baseline variables; general linear models with adjustment for baseline physical performance measures were used for change in physical performance over time, and Chi-squared and Fisher’s exact tests were used to analyse associations between baseline comorbidities and frailty categories.

The rate of change in physical performance was calculated for each individual using the slope of the regression line for time vs physical performance measures. For each individual, all available data points were included (i.e. participants were included if baseline and at least one follow-up measurement was available), and a slope value depicting rate of change per month of follow-up was generated. Rate of change was correlated with baseline physical performance for all measures; all correlations between rate of change and baseline biomarkers were therefore adjusted for the baseline value of the physical performance measure under test. Finally, multivariable linear regression was used to examine the relationship between baseline variables (including baseline physical performance) and rate of change in physical performance, again using forced entry of variables. Comorbidities were not included in multivariable analyses as they were likely to lie on the causal pathway between biochemical measures and physical performance in most cases and their inclusion would therefore prevent any effect of biochemical variables on physical performance from being apparent in the analyses. The sample size for this analysis (*n* = 300) was constrained by the numbers recruited to the BiCARB trial.

## Results

### Baseline details and frailty prevalence

Three hundred people were included in the analysis; baseline details are shown in Table 1. All participants were resident in their own homes; no participant was resident in a care home. Forty-two (14%) were non-frail, 148 (49%) were pre-frail, 86 (29%) were frail, and 24 (8%) had incomplete data that meant that a frailty score could not be calculated; these participants were still included in the frailty analyses as a separate group. Supplementary Table 2 shows how many individuals contributed physical performance data at each follow-up visit.

**Table 1:** Baseline details (*n* = 300)

Age (years) (SD)			74.0 (7.1)
Male sex (%)			214 (71)
eGFR category (%)	Stage 4		218 (72.7)
	Stage 5		82 (27.3)
Ethnicity (%)	White		287 (95.7)
	Far east asian		1 (0.3)
	Black african		1 (0.3)
	South asian		6 (2.0)
	Hispanic		1 (0.3)
	Other		4 (1.3)
Living status (%)	Living alone		91 (30.6)
	Not living alone		206 (69.4)
Hypertension (%)			264 (88.0)
Diabetes mellitus (%)			101 (33.7)
Ischaemic heart disease* (%)			57 (19.0)
Stroke (%)			28 (9.3)
Peripheral vascular disease (%)			24 (8.0)
Chronic heart failure (%)			24 (8.0)
Atrial fibrillation (%)			41 (13.7)
Body mass index (kg/m^2^) (SD)			28.6 (4.6)
Short physical performance battery score (SD)			8.1 (2.3)
Six-minute walk distance (m) (SD)			311 (134)
Maximum grip strength (kg) (SD)	Men		27.3 (8.2)
	Women		15.6 (4.6)
Cystatin C (mg/l) (SD)			3.1 (0.7)
Creatinine (μmol/l) (SD)			298 (102)
Bicarbonate (mmol/l) (SD)			21.3 (3.1)
eGFR (by MDRD4 equation, ml/min/1.73m^2^) (SD)			19.9 (6.8)
25-hydroxyvitamin D (nmol/l) (SD)			45 (27)
Median NT-pro-BNP (pg/ml) (IQR)			6413 (1647–10,295)
Albumin (g/l) (SD)			40 (5)
Haemoglobin (g/dl) (SD)			11.6 (1.6)
Phosphate (mmol/l) (SD)			1.3 (0.3)
*Frailty score components*			
Low grip strength (%)			156/300 (52.0)
Low gait speed (%)			159/296 (53.7)
Exhaustion (%)			151/287 (52.6)
BMI < 18.5 kg/m^2^ (%)			1/296 (0.3)
Low activity (%)			64/285 (22.5)
Frailty score (%)	0	Non-frail	42 (15.2)
	1	Pre-frail	82 (29.7)
	2		66 (23.9)
	3	Frail	59 (21.4)
	4		27 (9.8)
	5		0 (0)
	Score not calculable		24 (8.0)

All data are mean (SD) unless otherwise specified

*BMI* body mass index, *eGFR* estimated glomerular filtration rate, *25OHD* 25-hydroxyvitamin D, *NT-pro-BNP* N-terminal pro B-type natriuretic peptide

*Defined as previous myocardial infarction or coronary revascularisation

### Baseline associations with frailty

Table 2 shows the association between baseline biomarkers, adverse outcomes and different categories of frailty. Those living with frailty were older, with lower BMI, albumin, haemoglobin and 25OHD, and showed higher NT-pro-BNP concentrations than those who were non-frail, with a gradient between non-frail, pre-frail and frail evident for most of these variables. Biomarker measurements for participants where frailty could not be evaluated were similar to those with frailty. Trial dropout rates and death rates showed the expected relationship with frailty, being lowest in non-frail participants and highest in those with frailty or those people unable to complete frailty scoring. Falls rates were higher in those with frailty or pre-frailty than those who were non-frail. In contrast, adverse event rates were similar across all frailty categories. Baseline associations between cardiovascular comorbidities and frailty category are shown in Supplementary Table 3; peripheral vascular disease was the only comorbidity significantly associated with frailty in this analysis.

**Table 2:** Association of baseline variables and adverse outcomes with baseline frailty status

Variable		Non-frail (*n* = 42)	Pre-frail (*n* = 148)	Frail (*n* = 86)	Missing (*n* = 24)
Age (years) (SD)		70.7 (5.0)	73.3 (7.1)**	76.2 (7.3)**	75.6 (7.2)**
Cystatin C (mg/l) (SD)		2.9 (0.8)	3.1 (0.7)*	3.2 (0.7)**	3.2 (0.8)
Creatinine (μmol/l) (SD)		279 (93)	299 (93)	298 (115)	321 (121)
Bicarbonate (mmol/l) (SD)		21.0 (2.1)	21.2 (3.2)	21.4 (3.2)	21.1 (3.1)
BMI (kg/m^2^) (SD)		28.9 (3.8)	28.5 (4.0)	29.1 (5.8)	26.9 (3.3)*
eGFR by MDRD4 (ml/min/1.73m^2^) (SD)		20.9 (7.0)	20.0 (6.8)	19.7 (6.9)	18.2 (6.5)
25OHD (nmol/l) (SD)		55 (35)	45 (27)	42 (24)*	33 (19)*
Median NT-pro-BNP (pg/ml) (IQR)		4258 (407–6824)	6268 (2021–9553)	7501 (2368–12,223)**	7053 (988–11,257)
Albumin (g/l) (SD)		41 (5)	40 (4)	39 (5)*	38 (5)*
Haemoglobin (g/dl) (SD)		12.1 (1.5)	11.7 (1.7)	11.4 (1.4)*	11.2 (1.4)*
Phosphate (mmol/l) (SD)		1.3 (0.3)	1.3 (0.4)	1.2 (0.3)	1.4 (0.2)
*Adverse outcomes*					
Median number of adverse events (IQR)		2 (1 to 4)	2 (1 to 4)	3 (2 to 4)	2 (1 to 3)
Falls rate (per 1000 days)	Mean (SD)	0.27 (0.75)	1.63 (3.58)	1.46 (3.43)	1.58 (2.60)
	Median (IQR)	0 (0 to 0)	0 (0 to 2.22)**	0 (0 to 1.45)**	0 (0 to 2.87)*
Dropout by 12 months (%)		6 (14)	32 (22)	27 (31)*	9 (38)*
Death by 12 months (%)		0 (0)	7 (5)	6 (7)	2 (8)

*p < 0.05 **p < 0.01 vs non-frail

*BMI* body mass index, *eGFR* estimated glomerular filtration rate, *25OHD* 25-hydroxyvitamin D, *NT-pro-BNP* N-terminal pro B-type natriuretic peptide

**Table 3:** Baseline associations between biomarkers and physical performance measures

Variable		SPPB		6MWT		Grip (men)		Grip (women)	
		*r*	*p*	*r*	*p*	*r*	*p*	*r*	*p*
Age (years)		− 0.28	< 0.001	− 0.33	< 0.001	− 0.30	< 0.001	− 0.28	0.008
Cystatin C (mg/l)		− 0.10	0.13	− 0.19	0.002	− 0.17	0.02	− 0.22	0.07
Creatinine (μmol/l)		0.02	0.72	0.04	0.51	− 0.06	0.41	− 0.08	0.45
Bicarbonate (mmol/l)		− 0.11	0.06	0.01	0.86	0.02	0.73	− 0.05	0.67
BMI(kg/m^2^)		0.12	0.05	− 0.18	0.002	0.05	0.46	0.07	0.51
eGFR (ml/min/1.73m^2^)		0.01	0.83	0.04	0.50	0.06	0.42	0.06	0.61
25OHD (nmol/l)		0.17	0.02	0.25	< 0.001	0.10	0.20	0.26	0.05
NT-pro-BNP (pg/ml)*		− 0.27	< 0.001	− 0.32	< 0.001	− 0.31	< 0.001	− 0.12	0.35
Albumin (g/l)		0.14	0.02	0.20	< 0.001	0.12	0.10	0.10	0.35
Haemoglobin (g/dl)		0.15	0.014	0.22	< 0.001	0.12	0.08	0.10	0.38
Phosphate (mmol/l)		− 0.07	0.28	− 0.01	0.89	− 0.07	0.35	0.02	0.86
		Mean (SD)	*p*	Mean (m) (SD)	*p*				
Sex	Men	8.3 (2.2)	0.01	328 (129)	0.001	N/A	N/A	N/A	N/A
	Women	7.5 (2.4)		270 (137)		N/A	N/A	N/A	N/A

All Pearson’s correlations except for *Spearman’s rho

*SPPB* Short Physical Performance Battery,* 6WMD* Six-minute walk distance, *BMI* body mass index, *eGFR* estimated glomerular filtration rate, *25OHD* 25-hydroxyvitamin D, *NT-pro-BNP* N-terminal pro B-type natriuretic peptide

### Baseline correlations of physical performance

Table 3 shows the results of univariate associations with physical performance. Age, sex, BMI, NT-pro-BNP, albumin, and haemoglobin showed significant associations with SPPB score. For the 6MWD, age, cystatin C, BMI, NT-pro-BNP, 25-hydroxyvitamin D, albumin, and haemoglobin showed significant associations. For men, hand grip strength was associated with age, cystatin C, and BNP; different associations were seen for women with only age and 25-hydroxyvitamin D showing significant associations with grip strength. Univariate associations between baseline comorbidities and physical performance measures are given in Supplementary Table 4; diabetes mellitus, heart failure and atrial fibrillation were most consistently associated with worse physical performance. In multivariable analysis, only age and BMI were significantly associated with baseline SPPB; age, sex, BMI, NT-pro-BNP and 25OHD were all significantly associated with baseline 6MWD, but grip strength was significantly associated only with age (for men) and cystatin C (for women). Full details of the multivariable models are given in Supplementary Table 5.

### Associations with change in physical performance over time

Mean rates of change in physical performance were -0.02 (SD 0.25) points per month for SPPB, − 2.6 (SD 14.2) metres per month for 6MWD, and − 0.1 (SD 0.8) kg per month for grip strength. Table 4 shows associations between single baseline biomarkers and the rate of change (change per month) in physical performance measures over the follow-up period. For each measure of physical performance, higher baseline values correlated with a faster rate of fall in performance (regression to the mean) and so all results are adjusted for baseline physical performance. No significant association was seen between any baseline variable and the rate of change of any of the physical performance measures in univariate analyses. Univariate associations between baseline comorbidities and physical performance measures are given in Supplementary Table 6; no consistent association between baseline comorbidity and rate of change of physical performance measures was seen. Multivariable analyses are shown in Supplementary Table 7; only the baseline physical performance was associated with the rate of change of physical performance, together with baseline 25OHD concentration which was associated with the rate of change in 6MWD only.

**Table 4:** Univariate associations between baseline variables and rate of change in physical performance

Variable		SPPB		6MWD		Grip (men and women)	
		*r*	*p*	*r*	*P*	*r*	*p*
Age (years)		− 0.11	0.17	− 0.08	0.31	− 0.11	0.18
Cystatin C (mg/l)		− 0.01	0.95	− 0.02	0.85	0.00	0.98
Creatinine (μmol/l)		− 0.02	0.79	− 0.05	0.54	0.00	0.98
Bicarbonate (mmol/l)		− 0.05	0.53	0.09	0.28	0.05	0.52
BMI (kg/m^2^)		− 0.04	0.61	− 0.09	0.26	0.05	0.54
eGFR by MDRD4 (ml/min/1.73m^2^)		− 0.02	0.82	0.05	0.57	0.07	0.35
25OHD (nmol/l)		0.01	0.95	0.15	0.06	− 0.05	0.56
NT-pro-BNP (pg/ml)		− 0.08	0.32	− 0.09	0.28	0.00	0.98
Albumin (g/l)		− 0.01	0.87	0.06	0.46	− 0.11	0.16
Haemoglobin (g/dl)		− 0.05	0.55	0.11	0.16	0.05	0.54
Phosphate (mmol/l)		− 0.06	0.50	− 0.09	0.25	− 0.08	0.34
		Mean (points per month) (SD)	*p*	Mean (m per month) (SD)	*P*	Mean (Kg per month) (SD)	*p*
Sex	Men	− 0.03 (− 0.07 to 0.01)	0.21	− 3.0 (− 5.0 to − 1.0)	0.56	− 0.04 (− 0.15 to 0.07)	0.08
	Women	0.02 (− 0.05 to 0.08)		− 1.8 (− 5.1 to 1.5)		− 0.26 (− 0.46 to − 0.06)	

Partial correlations, adjusting for baseline value of physical performance measure in each analysis

*SPPB* Short Physical Performance Battery, *6WMD* Six-minute walk distance, *BMI* body mass index, *eGFR* estimated glomerular filtration rate, *25OHD* 25-hydroxyvitamin D, *NT-pro-BNP* N-terminal pro B-type natriuretic peptide

## Discussion

In this analysis, we found that frailty prevalence was higher in this group of participants with advanced CKD compared to the general older population. Most previous studies have focussed on patients on dialysis rather than on the much larger group of patients with advanced CKD who are not maintained on dialysis. The derived frailty categorisation was associated with adverse outcomes known to be related to the frailty syndrome, although total adverse events were similar in frequency across all frailty categories. Frailty was associated with greater age, lower 25OHD, albumin and haemoglobin concentrations and higher concentrations of cystatin C and NT-pro-BNP. The biomarkers studied did not consistently associate with either baseline measures of physical performance or with the rate of change in physical performance over time, and with the exception of some relationships with baseline six-minute walk distance (for 25OHD and NT-pro-BNP), the biomarkers studied were not independently associated with physical performance. Our results suggest that these markers may not be the right targets on which to focus interventions to improve frailty and physical performance in older people with advanced CKD, and that other approaches may be needed.

Our results are consistent with previous studies that have found a high prevalence of frailty in people with CKD. Given the age of our study sample (which is older than those enrolled in most CKD trials), this result is not unexpected but illustrates that it is not just patients with CKD on dialysis in whom frailty is very common. Impaired physical performance is a key component of the frailty syndrome, and participants in the trial had low 6MWD, low grip strength and low SPPB scores. The biochemical markers of CKD that we studied were not associated with differences in either physical performance at baseline or the trajectory of decline. This suggests that at least in this older population with advanced CKD, factors unrelated to the measured biochemical derangements, but caused by CKD may be more important in explaining impaired physical performance. It is important to note that the study population had high levels of comorbid disease [16], including conditions that would also be expected to impact on physical performance such as arthritis, cardiovascular disease and lung disease. It is, therefore, possible that these conditions were more important drivers of impaired physical performance than CKD itself. Another alternative is that aspects of deranged metabolism seen in CKD, but not measured directly in this study, could still be associated with impaired physical performance. It is unlikely that any association was affected by participants taking either bicarbonate or placebo since the main BiCARB trial findings did not show any significant differences in any outcome measures, including physical performance, between the intervention and placebo groups [16]. A number of uraemic toxins (e.g. indoxyl sulphate [27]) have been postulated to have direct effects on neurological and skeletal muscle function, and levels of these unmeasured toxins may not have been fully reflected by the markers (e.g. estimated GFR) employed in this analysis.

A systematic review published in 2018 [14] found cross-sectional associations between estimated GFR and both frailty and measures of physical performance including SPPB, 400 m walk, and self-reported disability. Equations using cystatin C rather than creatinine showed stronger associations, particularly with handgrip strength; this is likely to be due to the fact that low muscle mass (known to be associated with lower muscle strength) is associated with lower creatinine concentrations (and therefore a higher estimated GFR), leading to confounding of the relationship between eGFR and muscle mass. These studies examined older people with a range of kidney function from normal GFR through to kidney failure (GFR < 15 mL/min/1.73 m^2^). Although our study examined a narrower range of kidney function than these studies, our results were broadly comparable. Similarly, lower kidney function at baseline in longitudinal studies in this systematic review was associated with a higher risk of new disability or frailty in studies using cystatin C but this relationship was not always evident in studies using creatinine. Studies reported in this systematic review did not attempt to dissect out which aspects of metabolic derangement seen in CKD might be related to the presence of impaired physical performance. Additional data are expected on the relationship between kidney function and physical performance from the large Screening for Chronic Kidney Disease among Older People across Europe (SCOPE) study, which has enrolled 2500 people aged 75 and over [29]; initial results reinforce the confounding effect of muscle mass on the interpretation of eGFR equations.

Our analysis has a number of strengths; we were able to analyse data from older people with advanced CKD and with multiple morbidities, collected from multiple centres in a single country. This group of patients are typical of those seen in clinical practice but are less commonly enrolled in either trials or observational studies of kidney disease. Furthermore, we were able to analyse data from multiple follow-up timepoints up to 2 years after enrolment, enabling not only longitudinal analysis but analysis using the trajectory of change of physical performance over time. We were also able to measure both frailty (using a variant of a well-established frailty score) and physical performance in several complementary ways including measures of lower limb function (the SPPB), upper limb function (grip strength) and endurance/cardiorespiratory function (the six-minute walk distance). Finally, our focus on older people with CKD stages 4 and 5 is likely to maximise our ability to examine the impact of metabolic derangements associated with CKD. Such derangements (e.g. anaemia, derangements of bone and mineral metabolism, uraemic toxin retention) are thought to be less prominent in moderate CKD (GFR 30–59 mL/min/1.73 m^2^), and thus less of an effect on physical performance would be available to detect.

Some limitations also deserve comment. In line with the characteristics of the study population (advanced age, multimorbidity and frailty), dropout due to dialysis, illness or death was common during follow-up, increasing the amount of missing data and hence reducing our ability to detect modest associations. Despite the pragmatic design of the BiCARB trial, the analysis population was still drawn from a randomised controlled trial and are therefore likely to be fitter than the general population of older people with advanced CKD. In addition, the relatively low proportion of women, low ethnic diversity and UK origin of data, are all likely to limit the generalisability of the findings. It is also important to note that no adjustment was made for multiple testing; given the number of comparisons made in the analysis, it is possible that those associations that did reach significance could still be due to chance, and the findings require replication in other cohorts.

Although previous research has clearly demonstrated an association between CKD and both frailty and impaired physical performance, it has not been clear what aspects of CKD pathophysiology are most important in driving this association—nor indeed how much of the association is causal. Based on our results, interventions to improve physical performance and frailty in older people by targeting aspects of pathophysiology found in CKD may be of limited benefit. Of the domains tested, improving cardiovascular health, vitamin D supplementation and possibly weight loss for people with obesity may be worthy of further study as interventions to improve physical performance in randomised controlled trials for patients with CKD. The effects of vitamin D supplementation on physical performance in older people without CKD have been well studied; current analyses do not support a clinically important effect of supplementation on physical performance [29]. Similarly, bicarbonate therapy for metabolic acidosis does not appear to be effective at improving physical performance in older people, at least not at doses used currently in clinical practice [16]. Studies examining the relationship between physical performance and other modifiable factors (e.g. specific uraemic toxins) would, however, be informative; it is also possible that CKD pathophysiological derangements may be of more importance in driving frailty in younger people with CKD, who will tend to have less of the comorbidities that are also likely to limit physical performance independent of CKD. The greatest benefit may, however, come from interventions that target a broad range of diseases, either by pleiotropic effects (effects on a broad range of molecular targets) or by targeting specific fundamental biological pathways that underpin multiple diseases. Exercise training remains the archetype of such interventions, and remains the only intervention with solid evidence to ameliorate frailty and improve physical performance in older people [30], although other pathways such as inflammation and oxidative stress common to multiple diseases may also provide fruitful lines for future research.

## Supplementary Information

Below is the link to the electronic supplementary material.

Electronic supplementary material 1 (DOCX 29 kb)

## Data Availability

Deidentified, individual participant-level data are available to bona fide academic research teams, subject to submission of an outline of the purpose for which it will be used, and subject to approval by an independent Data Access Committee process hosted by the trial Sponsor (University of Dundee). For access, please contact the corresponding author or the Sponsor (TASCgovernance@dundee.ac.uk).

## Code availability

SPSS syntax is available on request from the corresponding author.
