# Supplementary material for: Associations between frailty, physical performance, and renal biomarkers in older people with advanced chronic kidney disease
Source: Eur Geriatr Med. 2021 Mar 17;12(5):943–52. doi: 10.1007/s41999-021-00478-4 (PMC8463514; doi:10.1007/s41999-021-00478-4)
Supplement: Supplementary file 1 — Electronic supplementary material 1 (DOCX 29 kb) [file 41999_2021_478_MOESM1_ESM.docx]

**Supplementary methods: Derivation of Frailty score**

Details of data sources and cut-offs used to derive a version of the Fried Frailty Score are given in Supplementary Table 1. Low strength was operationalised as handgrip below the revised European Working Group on Sarcopenia (EWGSOP) cut-offs [22]. Slow walk speed was operationalised as a SPPB score of 3 or less on the walking domain (equivalent to longer than 4.82 seconds, i.e. or a walk speed of 0.83m/s or less; very similar to the cut-off for low walk speed suggested by the EWGSOP criteria). For exhaustion, a vitality score of <55 on a 0-100 scale derived from the KDQoL score was used; this method has been employed previously using identical questions from the SF-36 health status questionnaire on which KDQoL is based [23-25]. Low weight was defined as BMI of <18.5kg/m^2^; data on weight loss before the baseline visit were not available, and low BMI has been used with success in previous variants of the Fried score [26]. Finally, low activity was operationalised as an answer of ‘Yes, limited a lot’ to the KDQoL question ‘Does your health now limit moderate activities such as moving a table, pushing a vacuum, bowling or playing golf’. This question has been used by other studies for operationalising the construct of low activity [25]. Domains were scored as 0 (criterion not met) or 1 (criterion met) and summed to produce a frailty score with a range of 0 to 5. A score of 0 was categorised as ‘non-frail’, 1-2 was ‘pre-frail’, and 3-5 was categorised as frail. To test whether frailty had the expected relationship with adverse outcomes, we analysed data on falls (normalised as number of falls per 1000 days), death and dropout within the first year of follow up, and number of adverse events per participant during the trial were extracted from the trial dataset and compared across frailty categories.

**Supplementary Table 1. Derivation of the Fried Frailty score in the BiCARB study population.**

| Component of Frailty | Definition |
| --- | --- |
| Weakness  EWGSOP2 cut off for max hand grip strength for Sarcopenia | Men = <27kg  Women = <16kg |
| Slowness  Modified EWGSOP2 cut off for low gait speed for Sarcopenia | <0.83m/s or 4m gait speed walk in >4.82s = frail |
| Exhaustion  Taken from KDQOL question 9 | Section A = ‘Did you feel full of pep’  Section E = ‘Did you have a lot of energy’  Section G = ‘Did you feel worn out’  Section I = ‘Did you feel tired’  Score averaged <55 = frail |
| Weight loss | Body mass index <18.5 kg/m^2^ is frail phenotype |
| Low activity  Taken from KDQOL question 3 | Section B = ‘Does your health now limit moderate activities such as moving a table, pushing a vacuum, bowling or playing golf’  Response of ‘Yes, limited a lot’ is frail phenotype |

EWGSOP: European Working Group on Sarcopenia in Older People. KDQOL: Kidney Disease Quality Of Life.

**Supplementary Table 2. Numbers undergoing each physical performance measure at each follow up timepoint**

|  | Number attending follow up visit, n (%) | SPPB, n (%) | 6MWD, n (%) | Grip strength, n (%) |
| --- | --- | --- | --- | --- |
| Baseline | 300 | 274 | 299 | 214 |
| 3 months | 274 | 243 | 262 | 198 |
| 6 months | 248 | 224 | 241 | 180 |
| 12 months | 220 | 187 | 210 | 157 |
| 24 months | 161 | 145 | 159 | 113 |

SPPB: Short Physical Performance Battery. 6WMD: Six minute walk distance

**Supplementary Table 3. Association of baseline comorbidities with baseline frailty category**

|  | Non-frail | Pre-frail | Frail | Missing | P* |
| --- | --- | --- | --- | --- | --- |
| Hypertension (%) | 37 (14.0) | 130 (49.2) | 76 (28.8) | 21 (8.0) | 0.99 |
| No hypertension (%) | 5 (13.9) | 18 (50.0) | 10 (27.8) | 3 (8.3) |  |
|  |  |  |  |  |  |
| Diabetes mellitus (%) | 11 (10.9) | 45 (44.6) | 35 (34.7) | 10 (9.9) | 0.23 |
| No diabetes mellitus (%) | 31 (15.6) | 103 (51.8) | 51 (25.6) | 14 (7.0) |  |
|  |  |  |  |  |  |
| Ischaemic heart disease (%) | 6 (10.5) | 26 (45.6) | 21 (36.8) | 4 (7.0) | 0.50 |
| No ischaemic heart disease (%) | 36 (14.8) | 122 (50.2) | 65 (26.7) | 20 (8.2) |  |
|  |  |  |  |  |  |
| Stroke (%) | 2 (7.1) | 11 (39.3) | 10 (35.7) | 5 (17.9) | 0.13 |
| No stroke (%) | 40 (14.7) | 137 (50.4) | 76 (27.9) | 19 (7.0) |  |
|  |  |  |  |  |  |
| Peripheral vascular disease (%) | 1 (4.2) | 10 (41.7) | 13 (54.2) | 0 (0) | 0.03 |
| No peripheral vascular disease (%) | 41 (14.9) | 138 (50.0) | 73 (26.4) | 24 (8.7) |  |
|  |  |  |  |  |  |
| Chronic heart failure (%) | 1 (4.2) | 11 (45.8) | 11 (45.8) | 1 (4.2) | 0.22 |
| No chronic heart failure (%) | 41 (14.9) | 137 (49.6) | 75 (27.2) | 23 (8.3) |  |
|  |  |  |  |  |  |
| Atrial fibrillation (%) | 1 (2.4) | 21 (51.2) | 16 (39.0) | 3 (7.3) | 0.06 |
| No atrial fibrillation (%) | 41 (15.8) | 127 (49.0) | 70 (27.0) | 21 (8.1) |  |

*Chi-squared test, or Fishers exact test (where cell size <5)

**Supplementary Table 4: Univariate association of comorbidities with physical performance**

|  | SPPB | | 6MW | | Grip (men) | | Grip (women) | |
| --- | --- | --- | --- | --- | --- | --- | --- | --- |
|  | Score (SD) | P* | Distance (m) (SD) | P* | Grip (kg) (SD) | P* | Grip (kg) (SD) | P* |
| Hypertension | 8.0 (2.3) | 0.18 | 310 (133) | 0.69 | 27.0 (8.3) | 0.13 | 15.8 (4.7) | 0.36 |
| No hypertension | 8.6 (2.0) |  | 319 (140) |  | 29.6 (7.7) |  | 14.3 (4.1) |  |
|  |  |  |  |  |  |  |  |  |
| Diabetes mellitus | 7.5 (2.3) | 0.005 | 265 (128) | <0.001 | 26.7 (10.1) | 0.46 | 14.4 (4.5) | 0.13 |
| No diabetes mellitus | 8.3 (2.2) |  | 334 (131) |  | 27.6 (7.0) |  | 16.0 (4.6) |  |
|  |  |  |  |  |  |  |  |  |
| Ischaemic heart disease | 7.5 (2.3) | 0.03 | 295 (142) | 0.31 | 26.4 (6.7) | 0.42 | 13.1 (4.1) | 0.07 |
| No ischaemic heart disease | 8.2 (2.2) |  | 315 (131) |  | 27.5 (8.6) |  | 15.9 (4.6) |  |
|  |  |  |  |  |  |  |  |  |
| Stroke | 8.2 (2.2) | 0.74 | 271 (139) | 0.10 | 26.0 (7.4) | 0.49 | 14.9 (5.2) | 0.63 |
| No stroke | 8.1 (2.3) |  | 315 (133) |  | 27.4 (8.3) |  | 15.7 (4.6) |  |
|  |  |  |  |  |  |  |  |  |
| Peripheral vascular disease | 7.3 (2.1) | 0.10 | 257 (128) | 0.04 | 26.1 (5.8) | 0.49 | 12.5 (3.0) | 0.18 |
| No peripheral vascular disease | 8.1 (2.3) |  | 315 (133) |  | 27.4 (8.4) |  | 15.7 (4.6) |  |
|  |  |  |  |  |  |  |  |  |
| Chronic heart failure | 7.6 (1.8) | 0.34 | 238 (118) | 0.005 | 23.4 (5.5) | 0.02 | 11.3 (4.0) | 0.10 |
| No chronic heart failure | 8.1 (2.3) |  | 317 (133) |  | 27.7 (8.4) |  | 15.7 (4.6) |  |
|  |  |  |  |  |  |  |  |  |
| Atrial fibrillation | 7.3 (2.2) | 0.03 | 267 (129) | 0.02 | 25.0 (6.9) | 0.07 | 13.5 (1.8) | 0.03 |
| No atrial fibrillation | 8.2 (2.2) |  | 318 (133) |  | 27.7 (8.4) |  | 15.7 (4.7) |  |

*Students t-test

**Supplementary Table 5. Multivariable associations with baseline physical performance measures**

|  | SPPB  (n=172) | | 6MWD  (n=184) | | Grip (M)  (n=138) | | Grip (F)  (n=47) | |
| --- | --- | --- | --- | --- | --- | --- | --- | --- |
|  | B | p | B | p | B | p | B | p |
| Age | -0.083 | **0.001** | -5.804 | **<0.001** | -0.262 | **0.02** | -0.132 | 0.21 |
| Sex | -0.531 | 0.18 | -61.695 | **0.002** | N/A | N/A | N/A | N/A |
| BMI | -0.079 | **0.05** | -8.331 | **<0.001** | -0.021 | 0.90 | 0.157 | 0.35 |
| Bicarbonate (mmol/L) | -0.039 | 0.49 | 2.978 | 0.32 | 0.154 | 0.54 | -0.373 | 0.19 |
| Cystatin C (mg/L) | 0.001 | 0.99 | -16.541 | 0.21 | -1.225 | 0.30 | -2.172 | **0.05** |
| 25OHD (nmol/L) | 0.009 | 0.15 | 0.677 | **0.04** | 0.034 | 0.21 | 0.028 | 0.39 |
| NT-pro-BNP (pg/ml) | <0.001 | 0.40 | -0.004 | **0.02** | <0.001 | 0.86 | <0.001 | 0.25 |
| Albumin (g/L) | 0.059 | 0.12 | 1.065 | 0.58 | 0.138 | 0.41 | 0.106 | 0.55 |
| Haemoglobin (g/dL) | 0.070 | 0.55 | 0.292 | 0.96 | -0.224 | 0.66 | 0.905 | 0.16 |
| Constant | 14.765 | <0.001 | 1004.082 | <0.001 | 43.442 | 0.004 | 18.537 | 0.29 |
| Adjusted R Squared | .108 | | .259 | | .026 | | .070 | |

SPPB: Short Physical Performance Battery. 6WMD: Six minute walk distance. BMI: Body mass index. eGFR: estimated Glomerular Filtration Rate. 25OHD: 25-hydroxyvitamin D. NT-pro-BNP: N-terminal pro B-type natriuretic peptide

**Supplementary Table 6. Univariate association between comorbidities and rate of change of physical performance measures**

|  | SPPB (points per month) | | 6MW | | Grip (men and women) | |
| --- | --- | --- | --- | --- | --- | --- |
|  | Points per month (SD) | P* | Metres per month (95% CI) | P* | Kg per month (SD) | P* |
| Hypertension | -0.02 (-0.05 to 0.02) | 0.68 | -2.9 (-4.7 to -1.1) | 0.42 | -0.10 (-0.20 to -0.01) | 0.83 |
| No hypertension | -0.04 (-0.12 to 0.05) |  | -0.8 (-5.7 to 4.1) |  | -0.07 (-033 to 0.18) |  |
|  |  |  |  |  |  |  |
| Diabetes mellitus | -0.02 (-0.08 to 0.04) | 1.00 | -4.4 (-7.5 to -1.4) | 0.17 | -0.17 (-0.32 to -0.01) | 0.32 |
| No diabetes mellitus | -0.02 (-0.06 to 0.02) |  | -1.8 (-3.8 to 0.2) |  | -0.07 (-0.18 to 0.04) |  |
|  |  |  |  |  |  |  |
| Ischaemic heart disease | -0.03 (-0.10 to 0.04) | 0.79 | -1.1 (-5.0 to 2.8) | 0.39 | -0.27 (-0.47 to -0.07) | 0.07 |
| No ischaemic heart disease | -0.02 (-0.05 to 0.02) |  | -3.0 (-4.9 to -1.1) |  | -0.06 (-0.16 to 0.04) |  |
|  |  |  |  |  |  |  |
| Stroke | 0.05 (-0.06 to 0.16) | 0.18 | -3.4 (-9.1 to 2.2) | 0.77 | 0.20 (-0.09 to 0.49) | 0.04 |
| No stroke | -0.03 (-0.06 to 0.01) |  | -2.6 (-4.3 to -0.8) |  | -0.13 (-0.22 to -0.04) |  |
|  |  |  |  |  |  |  |
| Peripheral vascular disease | -0.01 (-0.13 to 0.12) | 0.81 | -3.7 (-10.0 to 2.6) | 0.74 | 0.05 (-0.28 to 0.38) | 0.36 |
| No peripheral vascular disease | -0.02 (-0.05 to 0.01) |  | -2.6 (-4.3 to -0.8) |  | -0.11 (-0.20 to -0.02) |  |
|  |  |  |  |  |  |  |
| Chronic heart failure | -0.05 (-0.17 to 0.07) | 0.62 | -1.9 (-8.0 to 4.3) | 0.79 | -0.14 (-0.46 to 0.18) | 0.79 |
| No chronic heart failure | -0.02 (-0.05 to 0.01) |  | -2.7 (-4.5 to -0.9) |  | -0.10 (-0.19 to 0.00) |  |
|  |  |  |  |  |  |  |
| Atrial fibrillation | -0.07 (-0.15 to 0.01) | 0.20 | -5.8 (-10.4 to -1.3) | 0.13 | -0.05 (-0.29 to 0.18) | 0.67 |
| No atrial fibrillation | -0.01 (-0.05 to 0.02) |  | -2.1 (-4.0 to -0.3) |  | -0.11 (-0.20 to -0.01) |  |

*General linear model, adjusting for baseline physical performance value

**Supplementary Table 7. Multivariable associations with rate of change of physical performance measures**

|  | SPPB  (n=161) | | 6WD  (n=168) | | Grip (all)  (n=169) | |
| --- | --- | --- | --- | --- | --- | --- |
|  | B | p | B | p | B | p |
| Baseline value of measure | -0.038 | **<0.001** | -0.031 | **0.005** | -0.037 | **<0.001** |
| Age | -0.003 | 0.28 | -0.194 | 0.28 | -0.018 | 0.07 |
| Sex | 0.073 | 0.18 | -3.706 | 0.21 | -0.316 | 0.10 |
| BMI | -0.003 | 0.58 | -0.290 | 0.32 | 0.002 | 0.90 |
| Bicarbonate (mmol/L) | -0.003 | 0.64 | 0.465 | 0.26 | 0.011 | 0.64 |
| Cystatin C (mg/L) | 0.011 | 0.75 | -0.156 | 0.93 | -0.021 | 0.85 |
| 25OHD (nmol/L) | 0.000 | 0.98 | 0.101 | **0.03** | -0.002 | 0.43 |
| NT-pro-BNP (pg/ml) | -0.000 | 0.32 | -0.000 | 0.83 | -0.000 | 0.89 |
| Albumin (g/L) | -0.005 | 0.34 | 0.062 | 0.82 | -0.018 | 0.25 |
| Haemoglobin (g/dL) | -0.008 | 0.59 | -0.343 | 0.67 | 0.026 | 0.56 |
| Constant | 0.912 | 0.05 | 22.424 | 0.41 | 2.826 | 0.05 |
| Adjusted R squared | 0.064 | | 0.012 | | 0.075 | |

SPPB: Short Physical Performance Battery. 6WMD: Six minute walk distance. BMI: Body mass index. eGFR: estimated Glomerular Filtration Rate. 25OHD: 25-hydroxyvitamin D. NT-pro-BNP: N-terminal pro B-type natriuretic peptide
